# Supplementary material for: Combining RAS(ON) G12C-selective inhibitor with SHP2 inhibition sensitises lung tumours to immune checkpoint blockade
Source: Nat Commun. 2024 Sep 25;15:8146. doi: 10.1038/s41467-024-52324-3 (PMC11424635; doi:10.1038/s41467-024-52324-3)
Supplement: Supplementary file 1 — Supplementary Information [file 41467_2024_52324_MOESM1_ESM.pdf]

Supplementary Information

Combining RAS(ON) G12C-selective inhibitor with SHP2 inhibition sensitises lung tumours to immune checkpoint blockade

Panayiotis Anastasiou[1], Christopher Moore[1], Sareena Rana[1], Mona Tomaschko[1], Claire E Pillsbury[1], Andrea de Castro[1], Jesse Boumelha[1], Edurne Mugarza[1], Sophie de Carné Trécesson[1], Ania Mikolajczak[2], Cristina Blaj[3], Robert Goldstone[4], Jacqueline A.M. Smith[3], Elsa Quintana[3], Miriam Molina-Arcas[1,5] and Julian Downward[1,5]

[1] Oncogene Biology Laboratory, Francis Crick Institute, 1 Midland Road, London NW1 1AT, United Kingdom

[2] Experimental Histopathology, Francis Crick Institute, 1 Midland Road, London NW1 1AT, United Kingdom

[3]Revolution Medicines, Inc., Redwood City, CA 94063, USA

[4] Bioinformatics & Biostatistics Science Technology Platform, Francis Crick Institute, 1 Midland Road, London NW1 1AT, United Kingdom

[5] Correspondence: julian.downward@crick.ac.uk, miriam.molina@crick.ac.uk

Supplementary Fig. 1

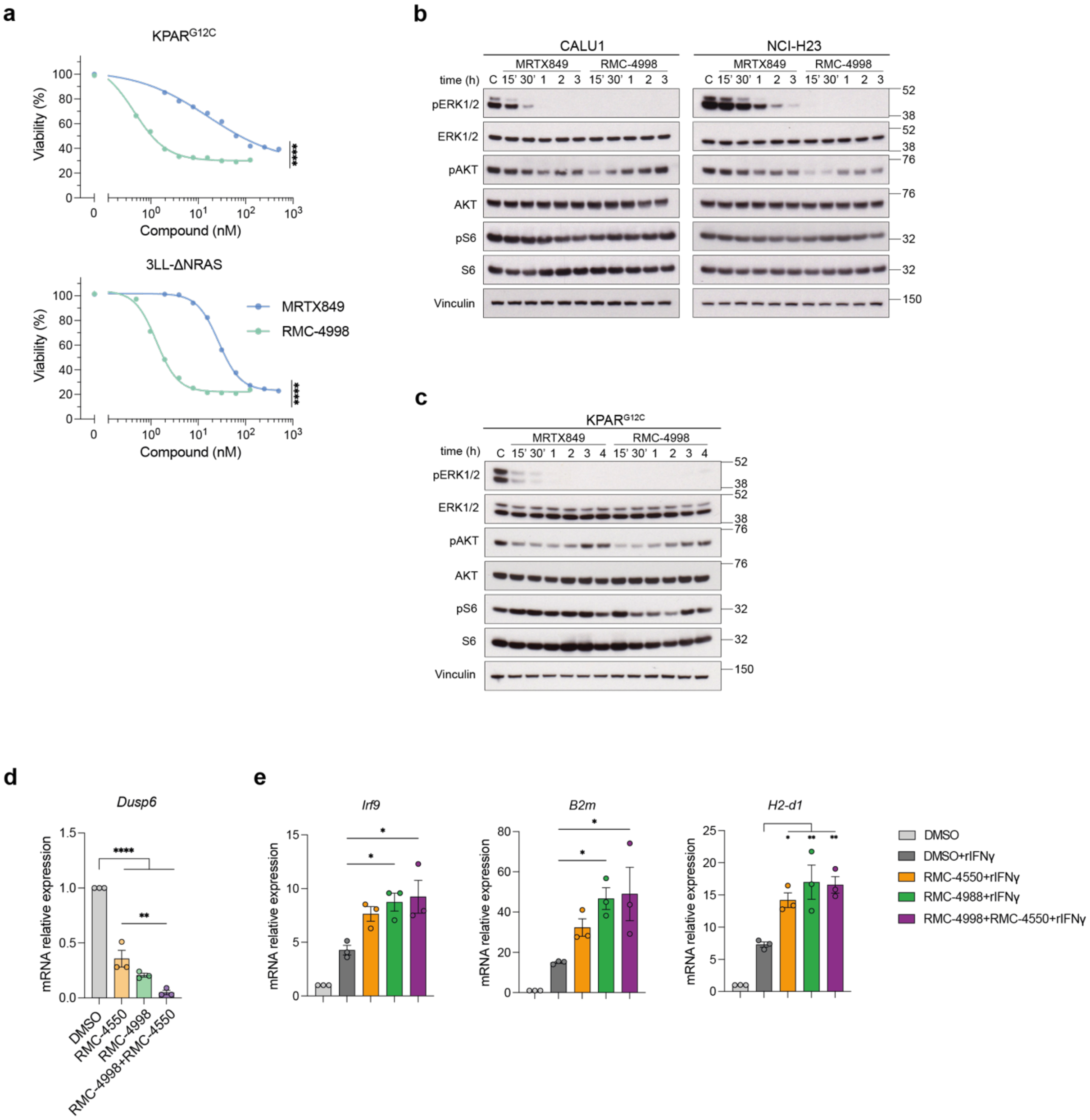

Supplementary Figure 1. SHP2 inhibitor RMC-4550 prevents adaptive response to the RAS(ON) G12C-selective inhibitor RMC-4998.

(a) Viability of mouse KRAS-mutant cell lines treated with serial dilutions of RMC-4998 or MRTX849 for 72 hours. Data are mean ± SEM of three independent experiments. Analysis was done using two-way ANOVA (****p < 0.0001).

(b) Western blot of human KRAS-mutant cell lines treated for 15 min, 30 min, 1, 2 or 3 hours with 100 nM MRTX849 or 100 nM RMC-4998. DMSO-treated cells were used a control (C). Blot is representative of two independent experiments.

(c) Western blot of mouse KRAS-mutant cell line treated for 15 min, 30 min, 1, 2 or 4 hours with 100 nM MRTX849 or 100 nM RMC-4998. Blot is representative of three independent experiments.

(d) qPCR analysis of Dusp6 in $KPAR^{G12C}$ cells treated for 24 hours with DMSO (n=3), 1 μM RMC-4550 (n=3), 100 nM RMC-4998 (n=3) or the combination (n=3). Data are mean ± SD of three independent experiments. Analysis was done using one-way ANOVA (**$p < 0.01$, ****$p < 0.0001$).

(e) qPCR analysis of IFN-induced genes in $KPAR^{G12C}$ cells treated for 24 hours with 1 μM RMC-4550, 100 nM RMC-4998 or the combination, in presence of 100 ng/ml IFNγ. DMSO treated cells are used as control. Data are mean ± SD of three independent experiments. Analysis was done using one-way ANOVA (*$p < 0.05$, **$p < 0.01$). Only significant comparisons are shown. The DMSO condition has only been compared to DMSO+rIFNγ. Source data are provided as a Source Data file.

Supplementary Fig. 2

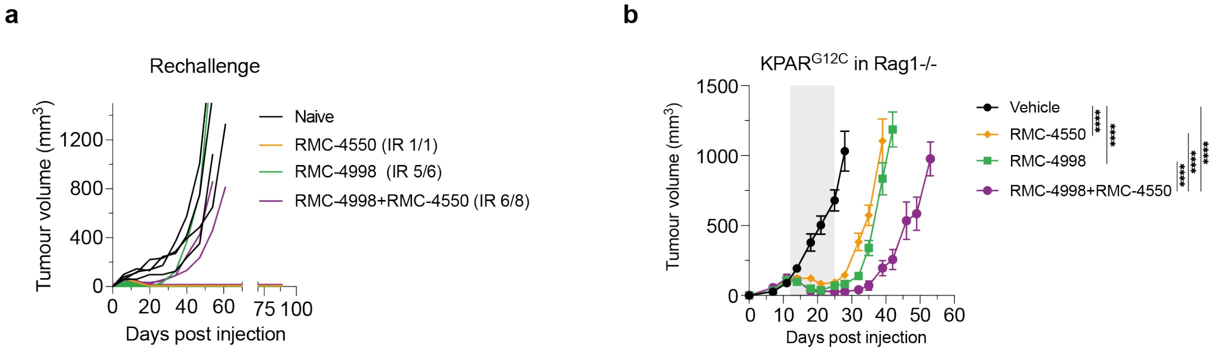

Supplementary Figure 2. Combination of the RAS(ON) G12C-selective inhibitor RMC-4998 with the SHP2 inhibitor RMC-4550 in immunogenic subcutaneous KPAR^{G12C} tumours.

(a) Mice in Fig. 2a that rejected the primary tumour were rechallenged on the opposite flank and tumour volume was measured. Number of mice that achieved immune rejections (IR) is indicated. Naïve mice of similar age were used as control. Legends indicate the treatment that the primary tumour received.

(b) Tumour growth of KPAR^{G12C} subcutaneous tumours grown in Rag1$^{-/-}$ mice treated daily for 2 weeks with 30 mg/kg RMC-4550 and/or 100 mg/kg RMC-4998. Vehicle (n=8), RMC-4550 (n=8), RMC-4998 (n=7), RMC-4998+RMC-4550 (n=8). Grey area indicates treatment period. Data are mean tumour volumes ± SEM. Analysis was performed using two-way ANOVA (****$p < 0.0001$). Individual tumour volumes are shown in Fig. 2c.

Source data are provided as a Source Data file.

Supplementary Fig. 3

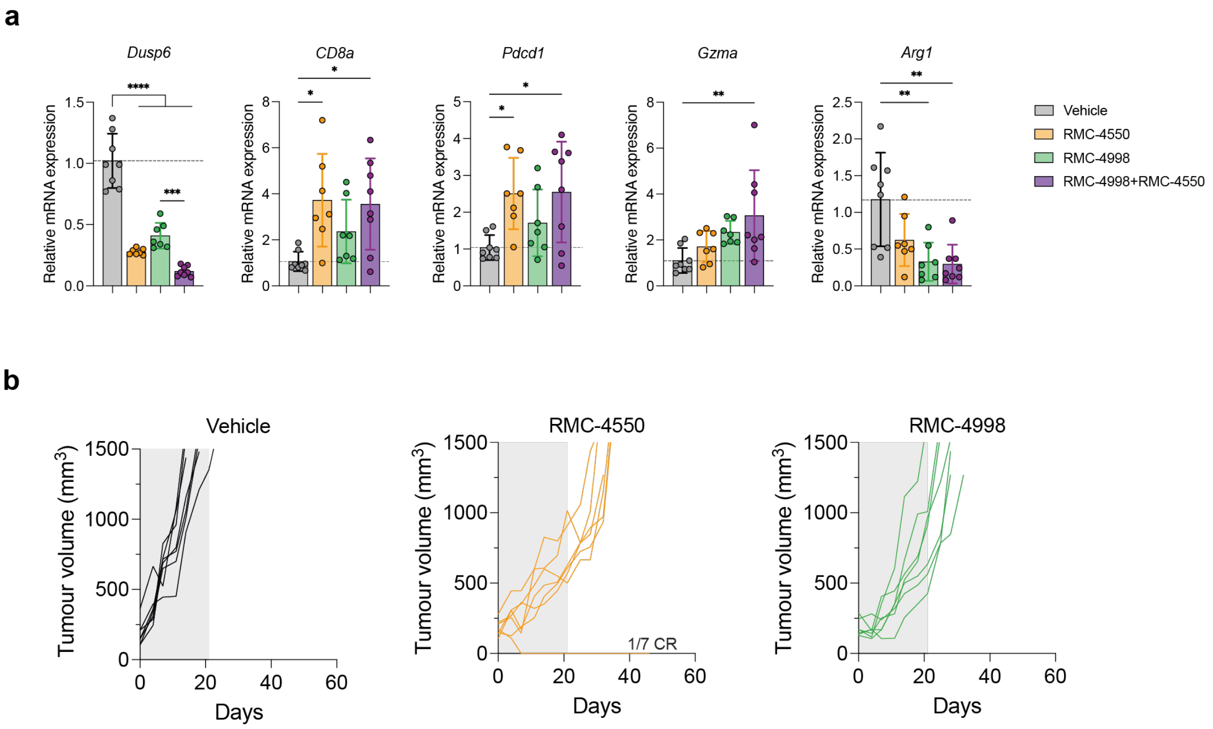

Supplementary Figure 3. Combination of the RAS(ON) G12C-selective inhibitor RMC-4998 with the SHP2 inhibitor RMC-4550 and/or anti-PD-1 in immunogenic KPAR^{G12C} tumours.

(a) qPCR analysis of immune genes of KPAR^{G12C} orthotopic lung tumours treated for 2 days with vehicle (n=8), 30 mg/kg RMC-4550 (n=7), 100 mg/kg RMC-4998 (n=7) or the combination (n=8). Data are mean values ± SD. Each dot represents one tumour, 2 tumours per mouse. Statistics were calculated using one-way ANOVA (*p < 0.05, **p < 0.01, ****p < 0.0001). Only significant comparisons are shown.

(b) Individual tumour volumes of mice in Fig. 3e. KPAR^{G12C}res subcutaneous tumours treated daily with vehicle (n=7), 100 mg/kg RMC-4998 (n=7) or 30 mg/kg RMC-4550 (n=7). Grey area indicates treatment period.

Source data are provided as a Source Data file.

Supplementary Fig. 4

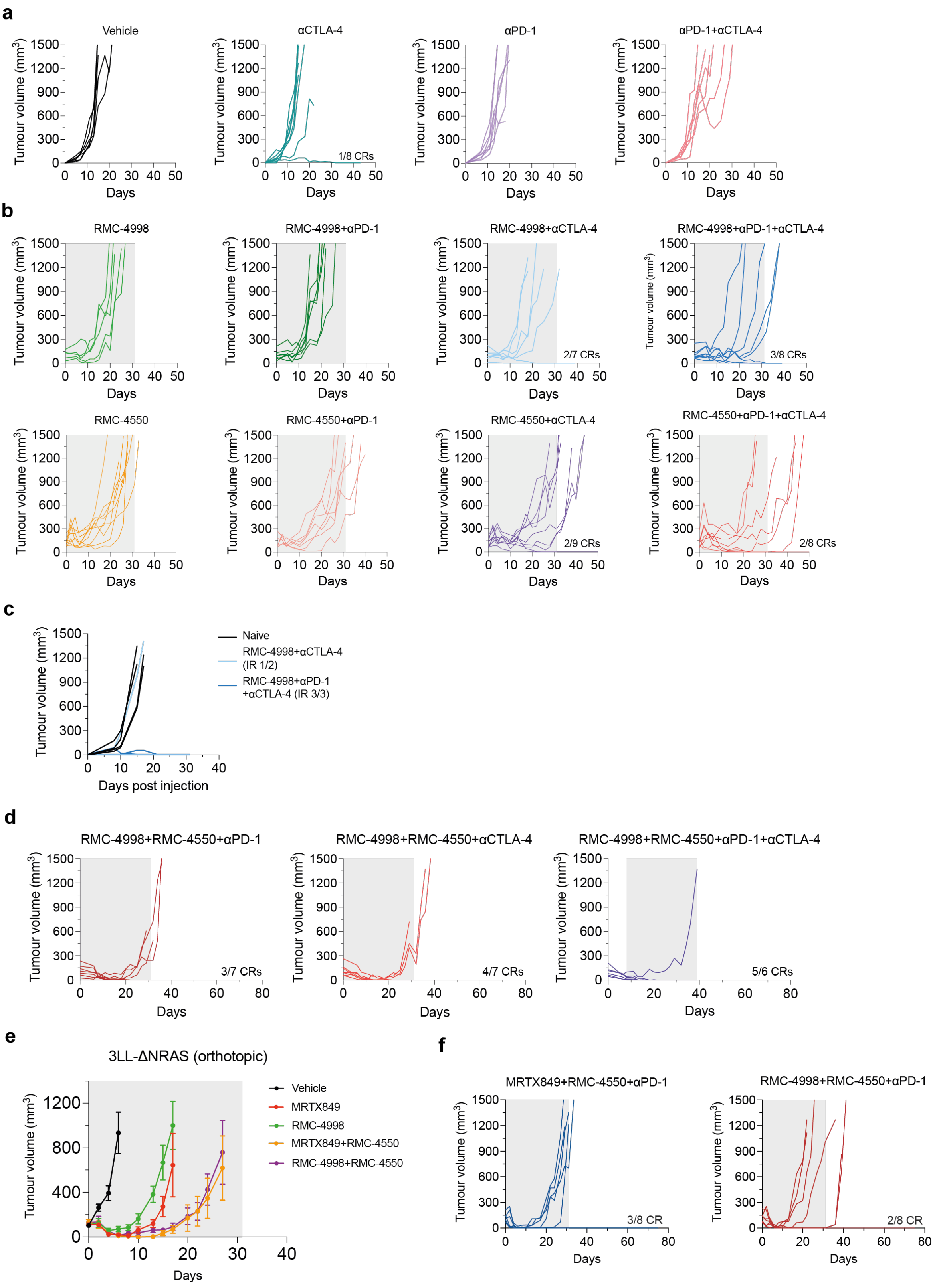

Supplementary Figure 4. Combination of RMC-4998 and RMC-4550 sensitises 3LL-ΔNRAS subcutaneous tumours to immunotherapies.

(a) Individual tumour volumes of mice in Fig. 3a. 3LL-ΔNRAS subcutaneous tumours treated with vehicle (n=6), 10 mg/kg anti-PD-1 (n=7), 5 mg/kg anti-CTLA-4 (n=8) or the combination (n=6). Antibodies were administered twice a week for two weeks. Grey area indicates treatment period. Same ICB doses and treatment schedules were administered in the other panels. Number of complete regressions (CR) is indicated.

(b) Individual tumour volumes of 3LL-ΔNRAS subcutaneous tumours treated with 100 mg/kg RMC-4998 or 30 mg/kg RMC-4550 in presence or absence of ICB. RMC-4998 (n=5), RMC-4998+anti-CTLA-4 (n=7), RMC-4998+anti-PD-1 (n=7), RMC-4998+anti-PD-1+anti-CTLA-4 (n=8), RMC-4550 (n=9), RMC-4550+anti-CTLA-4 (n=9), RMC-4550+anti-PD-1 (n=7), RMC-4550+anti-PD-1+anti-CTLA-4 (n=8). Grey area indicates treatment period. Number of complete regressions (CR) is indicated.

(c) Mice in top panel (b) that rejected the primary tumour were rechallenged on the opposite flank and tumour volume was measured. Number of mice that achieved immune rejections (IR) is indicated, RMC-4998+anti-CTLA-4 (n=2), RMC-4998+anti-PD-1+anti-CTLA-4 (n=3). Naïve mice (n=4) of similar age were used as control. Legends indicate the treatment that the primary tumour received.

(d) Individual tumour volumes of 3LL-ΔNRAS subcutaneous tumours mice treated with 100 mg/kg RMC-4998 plus 30 mg/kg RMC-4550 in presence or absence of ICB. Grey area indicates treatment period. Number of complete regressions (CR) is indicated.

(e) Tumour growth of 3LL-ΔNRAS subcutaneous tumours treated daily with 100mg/kg MRTX849 or 100 mg/kg RMC-4998 in presence or absence of 30 mg/kg RMC-4550. Vehicle (n=8), MRTX849 (n=7), RMC-4998 (n=8), MRTX849+RMC-4550 (n=7), RMC-4998+RMC-4550 (n=7). Grey area indicates treatment period. Data are mean tumour volumes ± SEM. Analysis was performed using two-way ANOVA test.

(f) Individual tumour volumes of mice treated with 100 mg/kg MRTX849 or 100 mg/kg RMC-4998 plus 30 mg/kg RMC-4550 and 10mg/kg anti-PD-1. Grey area indicates treatment period. Number of complete regressions (CR) is indicated.

Source data are provided as a Source Data file.

Supplementary Fig. 5

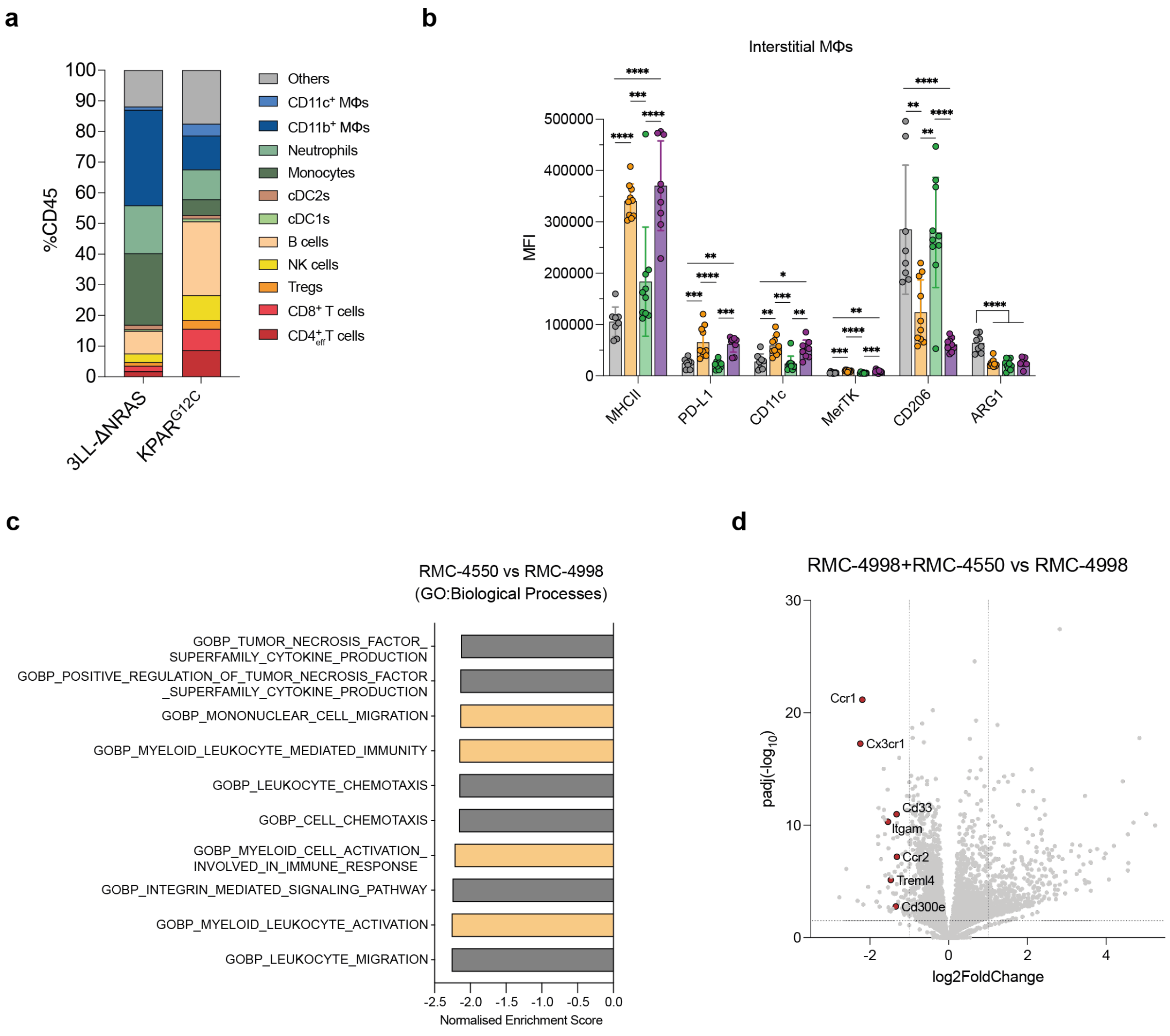

Supplementary Figure 5. RMC-4988 and RMC-4550 alter the myeloid cell-landscape in the TME of 3LL-ΔNRAS lung tumours.

(a) Flow cytometry immunophenotyping of untreated 3LL-ΔNRAS (n=9) and KPARG12C lung tumours (n=6).

(b) Individual mean fluorescence intensity of the samples plotted in the heatmap in Fig. 5c. 3LL-ΔNRAS lung tumours were treated for 8 days with 100 mg/kg RMC-4998, 30 mg/kg RMC-4550 or the combination. Vehicle (n=8), 100 mg/kg RMC-4998 (n=10), 30 mg/kg RMC-4550 (n=10) or the combination (n=9). Data are mean values ± SD. Each dot represents one independent biological mouse. Analysis was performed using one-way ANOVA test (*p < 0.05, **p < 0.01, ***p < 0.001, ****p < 0.0001). Only significant combinations are shown.

(c) Summary of top 10 significantly (FDR < 0.05) depleted pathways in RMC-4550 (n=4 mice, 2 individual tumours per mouse) treated tumours compared to RMC-4998 (n=4 mice, 2 individual tumours per mouse) treated tumours (MSigDB GO:Biological Processes).

(d) Volcano plot highlighting selected significant ($p < 0.05$) genes from 3LL-ΔNRAS lung tumours treated for 7 days with combination of 100 mg/kg RMC-4998 and 30 mg/kg RMC-4550 (n=4 mice, 2 individual tumours per mouse) vs 100 mg/kg RMC-4998 (n=4 mice, 2 individual tumours per mouse).
Source data are provided as a Source Data file.

Supplementary Fig. 6

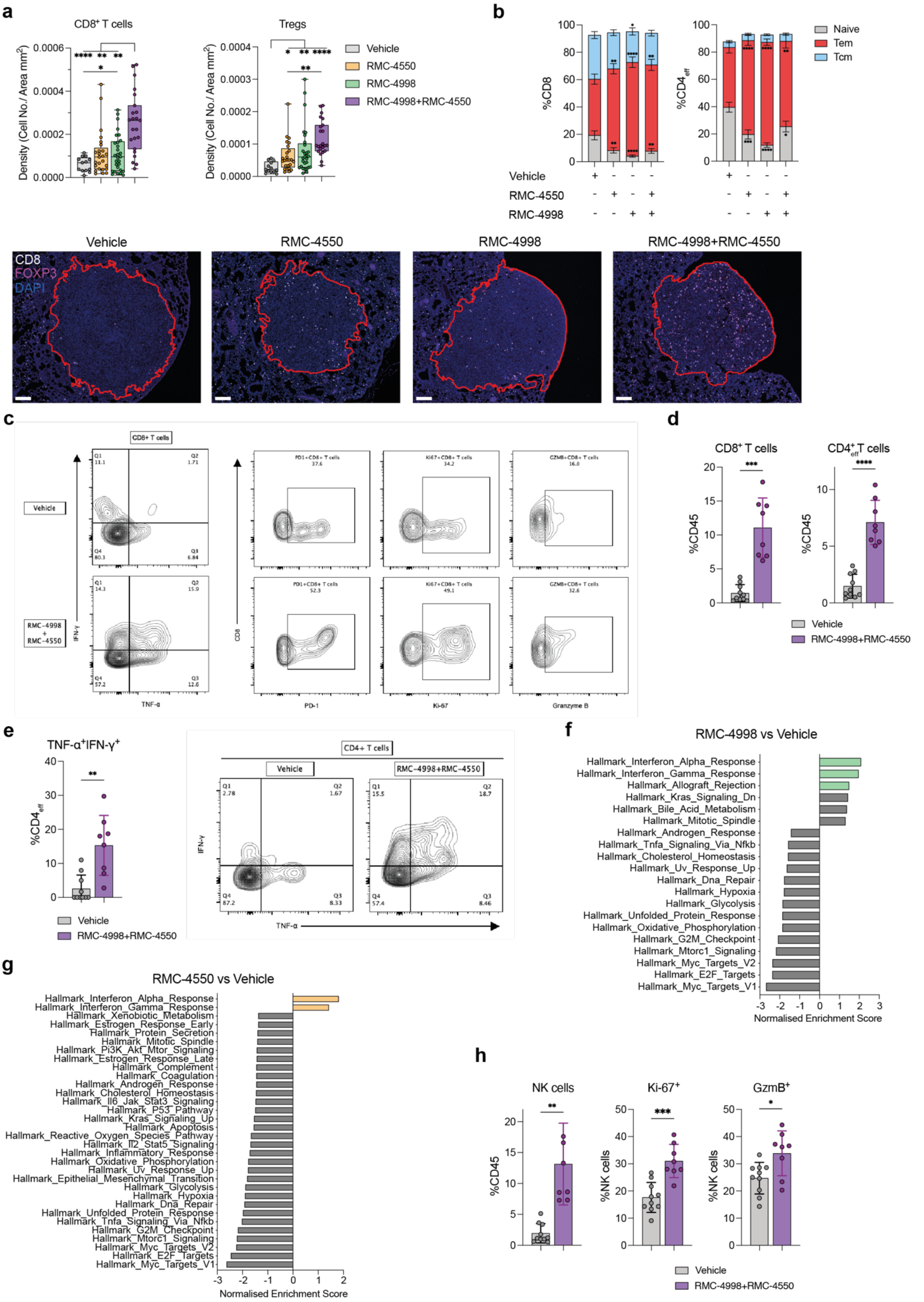

Supplementary Figure 6. RMC-4988 and RMC-4550 induce pan lymphocytic antitumour response in the TME of 3LL-ΔNRAS lung tumours.

(a) Quantification of immunofluorescence staining for CD8 T cells and Tregs (Foxp3) of 3LL-ΔNRAS orthotopic lung tumours treated for 7 days with 100 mg/kg RMC-4998, 30 mg/kg RMC-4550 or the combination. Vehicle (n=8), RMC-4550 (n=7), RMC-4998 (n=8), RMC-4998+RMC-4550 (n=6). Same doses were administered in the other panels. Data are shown as box and whisker with median. Box extends from the 25th to the 75th percentile and whiskers show min and max values. Each point is an individual tumour. Analysis was performed using one-way ANOVA. Bottom: Representative images of CD8 (white), Foxp3 (magenta) and DAPI (blue).

(b) Frequency of Naïve ($CD44^-CD62L^+$), Effector memory ($CD44^+CD62L^-$) and Central memory ($CD44^+CD62L^+$) $CD8^+$ and $CD4^+$ T cells from Fig. 4g. Data ± SEM of two independent experiments. Analysis was performed using one-way ANOVA.

(c) Representative flow plots of PD-1^+, Ki-67^+, TNF-α^+IFN-γ^+, $GzmB^+$ $CD8^+$ T cells from Fig. 6d.

(d, e) Frequency of $CD8^+$ from Fig. 6d and $CD4^+$ T cells (d) and frequency of TNF-α^+IFN-γ^+ $CD4^+$ T cells (e) along with representative flow plots. Data are mean values ± SD. Each dot represents a mouse. Analysis was performed using two-tailed Student's t-test.

(f, g) Summary of significantly (FDR < 0.05) down- or upregulated pathways in tumours treated with 100 mg/kg RMC-4998 (d) or 30 mg/kg RMC-4550 (e) compared vehicle treated tumours (MSigDB Hallmarks).

(h) Frequency of NK cells, Ki-67^+ and $GzmB^+$ NK cells in 3LL-ΔNRAS lung tumours treated for 7 days with combination of 100 mg/kg RMC-4998 and 30 mg/kg RMC-4550. Data are mean values ± SD. Each dot represents one mouse. Analysis was performed using two-tailed Student's t-test.

For all statistical analysis *p < 0.05, **p < 0.01, ***p < 0.001, ****p < 0.0001. Source data are provided as a Source Data file.

Supplementary Fig. 7

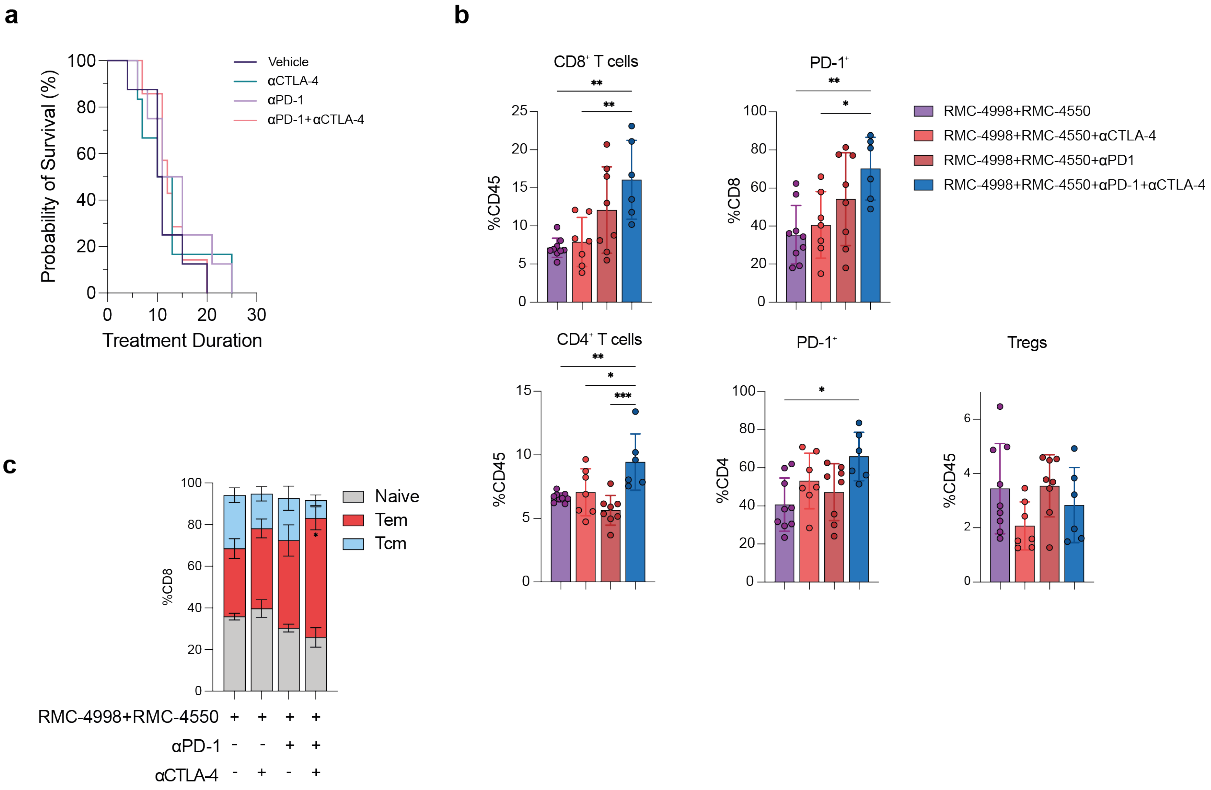

Supplementary Figure 7. RMC-4998 and RMC-4550 synergise with ICB an orthotopic immune-excluded anti-PD-1 resistant model of NSCLC.

(a) Survival of mice bearing 3LL-ΔNRAS orthotopic lung tumours treated with 4 doses of 10 mg/kg anti-PD-1 or 5 mg/kg anti-CTLA-4 or their combination within 2 weeks. Vehicle (n=8), anti-CTLA-4 (n=6), anti-PD-1 (n=6), anti-PD-1+anti-CTLA-4 (n=7).

(b) Frequency of $CD8^+$ T cells, $PD1^+$ $CD8^+$ T cells, $CD4^+$ T cells, $PD1^+$ $CD4^+$ T cells and Tregs in 3LL-ΔNRAS lung tumours treated for 7 days with the combination 100 mg/kg RMC-4998 and 30 mg/kg RMC-4550 in presence or absence of 10 mg/kg anti-CTLA-4 and/or 10 mg/kg anti-PD1 (twice). Data are mean values ± SD. Each dot represents a mouse. Analysis was done using one-way ANOVA (*p < 0.05, **p < 0.01, ***p < 0.001). Only significant comparisons are shown.

(c) Frequency of Naïve ($CD44^-CD62L^+$), Effector memory ($CD44^+CD62L^-$) and Central memory ($CD44^+CD62L^+$) $CD8^+$ T cells from panel (b). Data are mean values ± SEM. Analysis was done using one-way ANOVA (*p < 0.05).

Source data are provided as a Source Data file.

Supplementary Fig. 8

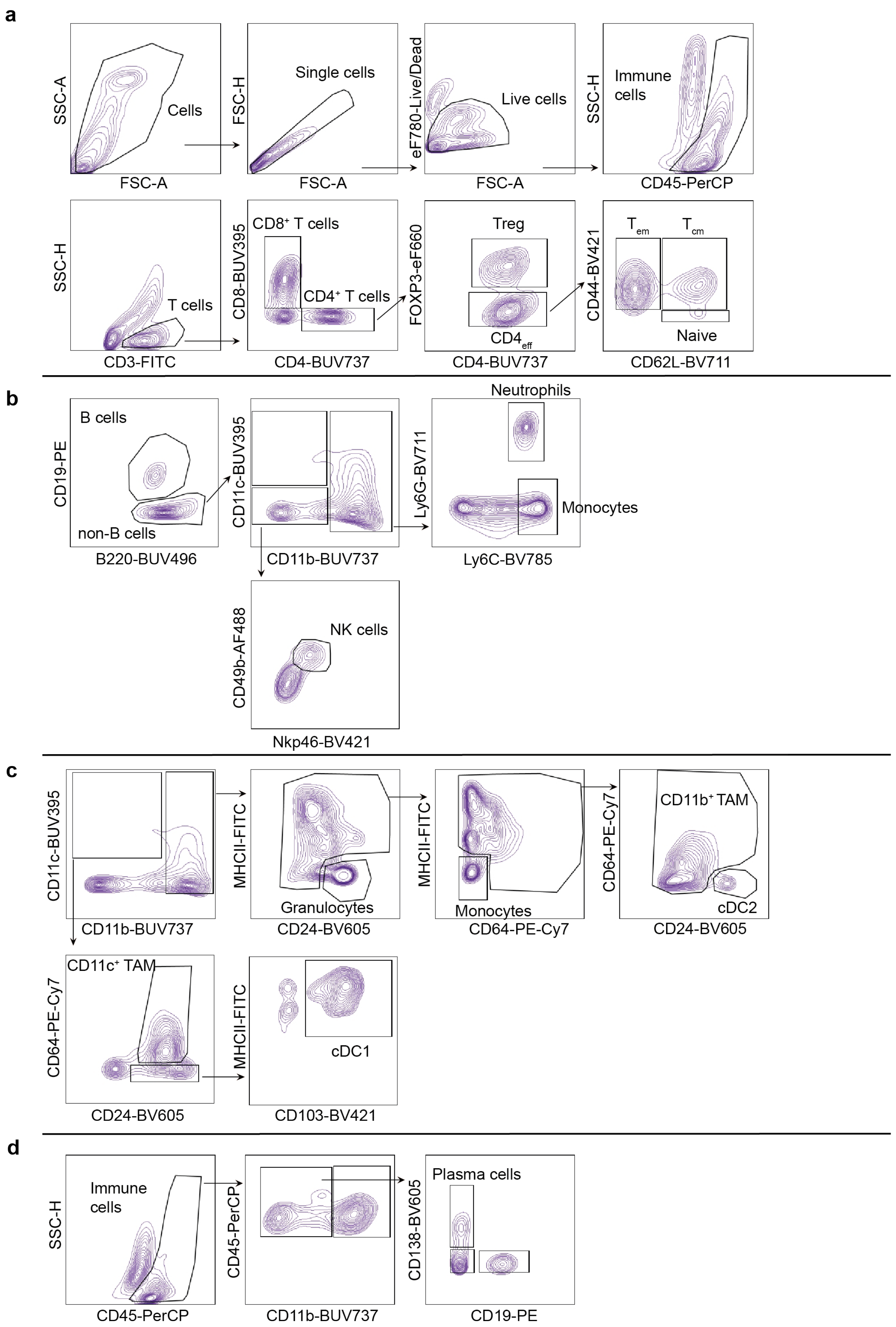

Supplementary Figure 8. Conventional flow cytometry gating strategies.

(a) Gating flow strategy for acquiring single, live, $CD45^+$ cells and further gating of T cells populations for Fig. 6 and S6.

(b-d) Gating flow strategy of $CD45^+$ cells to acquire B cells, monocytes, neutrophils and NK cells (b), $CD11b^+$ TAMs, $CD11c^+$ TAMs, cDC1s and cDC2s (c) and plasma cells (d).

Supplementary Fig. 9

a

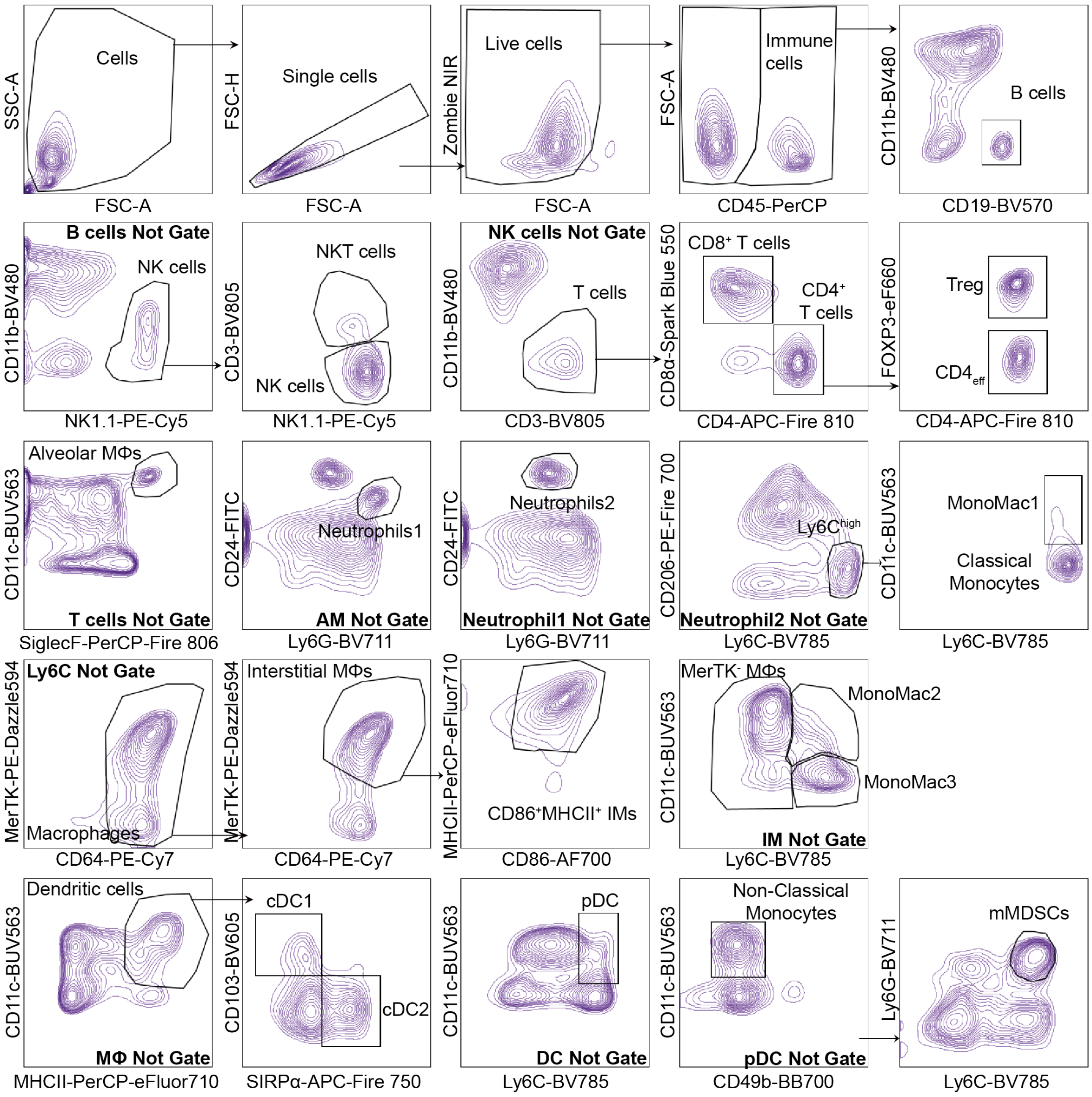

Supplementary Figure 9. Spectral flow cytometry gating strategy.

(a) Gating flow strategy for acquiring immune cells shown in Fig. 5f.

Supplementary Table 1. Antibodies used for conventional flow cytometry.

Protein	Clone	Fluorophore	Reference	Source	Dilution
CD103	2E7	BV421	121421	BioLegend	1:200
CD11b	M1/70	BUV737	612801	BD Horizon	1:200
CD11c	HL3	BUV395	564080	BD Horizon	1:80
CD138/Syndecan-1	281-2	BV605	142531	BioLegend	1:100
CD19	1D3/CD19	PE	152407	BioLegend	1:80
CD206 (MMR)	C068C2	BV711	141727	BioLegend	1:200
CD24	M1/69	BV605	101827	BioLegend	1:400
CD3	17A2	FITC	100204	BioLegend	1:100
CD335 (Nkp46)	29A1.4	BV421	137611	Biolegened	1:40
CD4	GK1.5	BUV737	612761	BD Horizon	1:200
CD44	IM7	BV421	103040	BioLegend	1:80
CD45	30-F11	PerCP	103129	BioLegend	1:600
CD45R/B220	RA3-6B2	BUV496	612950	BD Horizon	1:200
CD49b	DX5	AF488	108913	BioLegend	1:120
CD62L	MEL-14	BV711	104445	BioLegend	1:400
CD8a	53-6.7	BUV395	563786	BD Horizon	1:200
FcgRI (CD64)	X54-5/7.1	PE-Cy7	139313	BioLegend	1:200
Foxp3	FJK-16s	eF660	50-5773-82	eBioscience	1:80
Granzyme B	QA16A02	PE	372207	BioLegend	1:50
IFN-γ	XMG1.2	BV711	564336	BD Horizon	1:50
Ki-67	B56	BV786	563756	BD Horizon	1:100
Ly6C	HK1.4	BV785	128041	Biolegened	1:80
Ly6G	1A8	BV711	127643	Biolegened	1:80
MHCII (I-A/I-E)	M5/114.15.2	FITC	107605	BioLegend	1:800
PD-1 (CD279)	29F.1A12	BV785	135225	BioLegend	1:160
PD-L1 (CD274)	10F.9G2	PE	124308	BioLegend	1:80
TNF-α	MP6-XT22	BV605	506329	BioLegend	1:50
CD16/32	2.4G2	-	AB_2687830	BDBiosciences	1:50

Supplementary Table 2. Antibodies used for western bots.

Protein	Clone	Reference	Source	Dilution
Anti-S6	54D2	2238583	Cell Signalling	1:1000
Anti-p-S6 (Ser235/236)	Polyclonal	331679	Cell Signalling	1:1000
Anti-Erk1/2	3A7	10695739	Cell signalling	1:1000
Anti-p-Erk1/2 (Thr202/Tyr204)	9101	331646	Cell Signalling	1:1000
Anti-Akt	40D4	1147620	Cell Signalling	1:1000
Anti-p-Akt (Ser473)	D9E	2315049	Cell Signalling	1:1000
Anti-Vinculin	VIN-11-5	2877646	Sigma	1:2000

Supplementary Table 3. Antibodies used for spectral flow cytometry.

Protein	Clone	Fluorophore	Reference	Source	Dilution
CD45	30-F11	PerCP	103129	BioLegend	1:200
CD19	6D5	BV570	115535	BioLegend	1:200
CD11b	M1/70	BV480	566117	BD Biosciences	1:400
NK1.1	PK136	PE/Cy5	108715	BioLegend	1:200
CD3	17A2	BUV805	569192	BD Biosciences	1:150
CD8α	53-6.7	Spark Blue 550	100779	BioLegend	1:200
CD4	GK1.5	APC/Fire 810	100479	BioLegend	1:200
FOXP3	FJK-16	eFluor660	50-5773-82	eBioscience	1:200
CD11c	N418	BUV563	749040	BD Biosciences	1:150
Siglec-F	S17007L	PerCP/Fire 806	155535	BioLegend	1:200
CD24	M1/69	FITC	101805	BioLegend	1:400
Ly6G	1A8	BV711	127643	BioLegend	1:200
CD206	C068C2	PE/Fire 700	141741	BioLegend	1:400
Ly6C	HK1.4	BV785	128041	BioLegend	1:200
MerTK	2B10C42	PE/Dazzle 594	151523	BioLegend	1:150
CD64	X54-5/7.1	PE/Cy7	139313	BioLegend	1:200
MHCII	M5/114.15.2	PerCP-eFluor710	46-5321-82	eBioscience	1:600
CD86	GL-1	AF-700	105023	BioLegend	1:400
CD103	2E7	BV605	121433	BioLegend	1:100
SIRPα	P84	APC/Fire750	144029	BioLegend	1:200
CD49b	DX5	BB700	568015	BD Biosciences	1:200
CD107α	1D4B	BUV395	565533	BD Biosciences	1:150
PD-L1	10F.9G2	PE	124307	BioLegend	1:400
Arginase 1	A1exF5	eFluor450	48-3697-82	eBioscience	1:200
Ki-67	SolA15	BUV615	366-5698-82	eBioscience	1:600
TIM3	RMT2-23	BUV661	753149	BD Biosciences	1:100
CD62L	MEL-14	BUV737	612833	BD Biosciences	1:200
CD44	IM7	BV421	103039	BioLegend	1:200
CD69	H1.2F3	BV510	104531	BioLegend	1:150
B220	RA3-6B2	BUV496	612950	BD Biosciences	1:400
PD-1	J43	BV650	569506	BD Biosciences	1:200
H-2Kb	AF6-88.5.5.3	NovaFluorBlue610/70S	17836316	Invitrogen	1:200
CD71	C2	RB780	755614	BD Biosciences	1:400
CTLA-4	UC10-4B9	PE/Fire810	106335	BioLegend	1:150

Supplementary Table 4. qPCR primers.

Gene	Forward	Reverse
Arg1	ATGGGCAACCTGTGTCCTTT	TTCCCCAGGGTCTACGTCTC
B2m	TCTCACTGACCGGCCTGTAT	ATTTCAATGTGAGGCGGGTG
Cd274	CGCCACAGCGAATGATGTTT	AGGATGTGTTGCAGGCAGTT
Cd8a	TCAGTGAAGGGGACCGGATT	CTTCCTGTCTGACTAGCGGC
Dusp6	GAGCCAAAACCTGTCCCAGT	GTGACAGAGCGGCTGATACC
Gapdh	CAAGCTCATTTCCTGGTATGACA	GGATAGGGCCTCTCTTGCTC
Gzmb	Quantitect QT00114590 (Qiagen)	
H2-d1	Quantitect QT01657761 (Qiagen)	
Hsp90	AGATTCCACTAACCGACGCC	TGCTCTTTGCTCTCACCAGT
Ifng	ACAGCAAGGCGAAAAAGGATG	TGGTGGACCACTCGGATGA
Irf1	GACCCTGGCTAGAGATGCAG	CTCCGGAACAGACAGGCATC
Irf7	GCGTACCCTGGAAGCATTTC	GCACAGCGGAAGTTGGTCT
Irf9	GCCGAGTGGTGGGTAAGAC	GCAAAGGCGCTGAACAAAGAG
Nos2	GGAATGGAGACTGTCCCAGC	CGATGTCATGAGCAAAGGCG
Pdcd1	ACCCTGGTCATTCACTTGGG	CATTTGCTCCCTCTGACACTG
Prf1	TGGAGGTTTTTGTACCAGGC	TAGCCAATTTTGCAGCTGAG
Sdha	TCGACAGGGGAATGGTTTGG	TCATACTCATCGACCCGCAC
