# Supplementary material for: Combining RAS(ON) G12C-selective inhibitor with SHP2 inhibition sensitises lung tumours to immune checkpoint blockade
Source: Nat Commun. 2024 Sep 25;15:8146. doi: 10.1038/s41467-024-52324-3 (PMC11424635; doi:10.1038/s41467-024-52324-3)
Supplement: Supplementary file 3 — Reporting Summary [file 41467_2024_52324_MOESM3_ESM.pdf]

nature portfolio

Corresponding author(s): Julian Downward

Last updated by author(s): Aug 15, 2024

Reporting Summary

Nature Portfolio wishes to improve the reproducibility of the work that we publish. This form provides structure for consistency and transparency in reporting. For further information on Nature Portfolio policies, see our Editorial Policies and the Editorial Policy Checklist.

Statistics

For all statistical analyses, confirm that the following items are present in the figure legend, table legend, main text, or Methods section.

n/a	Confirmed	
☐	☒	The exact sample size (n) for each experimental group/condition, given as a discrete number and unit of measurement
☐	☒	A statement on whether measurements were taken from distinct samples or whether the same sample was measured repeatedly
☐	☒	The statistical test(s) used AND whether they are one- or two-sided<br>*Only common tests should be described solely by name; describe more complex techniques in the Methods section.*
☒	☐	A description of all covariates tested
☒	☐	A description of any assumptions or corrections, such as tests of normality and adjustment for multiple comparisons
☐	☒	A full description of the statistical parameters including central tendency (e.g. means) or other basic estimates (e.g. regression coefficient) AND variation (e.g. standard deviation) or associated estimates of uncertainty (e.g. confidence intervals)
☐	☒	For null hypothesis testing, the test statistic (e.g. F, t, r) with confidence intervals, effect sizes, degrees of freedom and P value noted<br>*Give P values as exact values whenever suitable.*
☒	☐	For Bayesian analysis, information on the choice of priors and Markov chain Monte Carlo settings
☒	☐	For hierarchical and complex designs, identification of the appropriate level for tests and full reporting of outcomes
☒	☐	Estimates of effect sizes (e.g. Cohen's d, Pearson's r), indicating how they were calculated

Our web collection on statistics for biologists contains articles on many of the points above.

Software and code

Policy information about availability of computer code

Data collection	Fortessa Symphony A5 for FACS<br>Quantum GX2 micro-CT imaging system (Perkin Elmer) for Ct scans<br>Illumina NovaSeq 6000 system for RNA sequencing<br>Spectral Analyser Aurora (Cytek) for Spectral<br>PhenoImager HT (formerly Vectra Polaris) for Immunofluorescence
Data analysis	FlowJo v10 (Tree Star Inc) for flow cytometry<br>Analyse (AnalyzeDirect) for Ct scan analysis<br>GraphPad Prism 10 Software for statistical analysis<br>R (versions 3.6.1.4.0.0)<br>RNAseq analysis: nfcore/rnaseq pipeline with STAR and RSEM, R package DESeq2, R package fgsea, R package msigdbr.<br>QuPath 0.5.0x64

For manuscripts utilizing custom algorithms or software that are central to the research but not yet described in published literature, software must be made available to editors and reviewers. We strongly encourage code deposition in a community repository (e.g. GitHub). See the Nature Portfolio guidelines for submitting code & software for further information.

Data

Policy information about availability of data

All manuscripts must include a data availability statement. This statement should provide the following information, where applicable:

- Accession codes, unique identifiers, or web links for publicly available datasets
- A description of any restrictions on data availability
- For clinical datasets or third party data, please ensure that the statement adheres to our policy

RNA-seq data has been deposited at Gene Expression Omnibus under accession number GSE254755

Research involving human participants, their data, or biological material

Policy information about studies with human participants or human data. See also policy information about sex, gender (identity/presentation), and sexual orientation and race, ethnicity and racism.

Reporting on sex and gender	N/A
Reporting on race, ethnicity, or other socially relevant groupings	N/A
Population characteristics	N/A
Recruitment	N/A
Ethics oversight	N/A

Note that full information on the approval of the study protocol must also be provided in the manuscript.

Field-specific reporting

Please select the one below that is the best fit for your research. If you are not sure, read the appropriate sections before making your selection.

☒ Life sciences ☐ Behavioural & social sciences ☐ Ecological, evolutionary & environmental sciences

For a reference copy of the document with all sections, see nature.com/documents/nr-reporting-summary-flat.pdf

Life sciences study design

All studies must disclose on these points even when the disclosure is negative.

Sample size	No statistical methods were used to predetermine sample size.<br>For in vivo experiments, group sizes were determined based on previous experience with the models used (Mugarza et al., Science Adv., 2022). The number of repeats were determined by the balance between statistical significance and reduction of animal use.<br>qPCR studies used a minimum 4 mice, 2 tumours per mouse, per group. For in vivo FACS we used minimum 6 mice per group. For survivals and subcutaneous tumour growth we used minimum 5 mice per group. In vitro experiments were carried out at least twice (2 independent experiments).
Data exclusions	In the 3LL vivo FACS experiments in Fig. 6 one lung tumour sample from RMC-4998 treated mice was excluded due to being comprised of 52.5% of B cells and 27.7% T cells and therefore was potentially a lymph node.
Replication	Figure legends indicate the number of repetitions. Some survival in vivo experiments were not performed twice due the high numbers of mice being used in one experiment. All attempts of repetition were successful.
Randomization	Mice were randomised into groups so that tumour burden would be equal between groups before starting treatment.
Blinding	Samples from mice were assigned a number so that during data analysis the treatment would be unknown. Exclusion of sample in previous section was done without knowing the treatment. For survival experiments, investigators were not blinded to group allocation as we were cautious for long term adverse effects of the triple and quadruple combinations.

Reporting for specific materials, systems and methods

We require information from authors about some types of materials, experimental systems and methods used in many studies. Here, indicate whether each material, system or method listed is relevant to your study. If you are not sure if a list item applies to your research, read the appropriate section before selecting a response.

Materials & experimental systems

n/a	Involved in the study	
	☒	Antibodies
	☒	Eukaryotic cell lines
☒		Palaeontology and archaeology
	☒	Animals and other organisms
☒		Clinical data
☒		Dual use research of concern
☒		Plants

Methods

n/a	Involved in the study	
☒		ChIP-seq
	☒	Flow cytometry
☒		MRI-based neuroimaging

Antibodies

Antibodies used

Flow cytometry (conventional)
CCD103 2E7 BV421 121421 BioLegend 1:200
CD11b M1/70 BUV737 612801 BD Horizon 1:200
CD11c HL3 BUV395 564080 BD Horizon 1:80
CD138/Syndecan-1 281-2 BV605 142531 BioLegend 1:100
CD19 1D3/CD19 PE 152407 BioLegend 1:80
CD206 (MMR) C068C2 BV711 141727 BioLegend 1:200
CD24 M1/69 BV605 101827 BioLegend 1:400
CD3 17A2 FITC 100204 BioLegend 1:100
CD335 (Nkp46) 29A1.4 BV421 137611 Biolegened 1:40
CD4 GK1.5 BUV737 612761 BD Horizon 1:200
CD44 IM7 BV421 103040 BioLegend 1:80
CD45 30-F11 PerCP 103129 BioLegend 1:600
CD45R/B220 RA3-6B2 BUV496 612950 BD Horizon 1:200
CD49b DX5 AF488 108913 BioLegend 1:120
CD62L MEL-14 BV711 104445 BioLegend 1:400
CD8a 53-6.7 BUV395 563786 BD Horizon 1:200
FcgRI (CD64) X54-5/7.1 PE-Cy7 139313 BioLegend 1:200
Foxp3 FJK-16s eF660 50-5773-82 eBioscience 1:80
Granzyme B QA16A02 PE 372207 BioLegend 1:50
IFN-γ XMG1.2 BV711 564336 BD Horizon 1:50
Ki-67 B56 BV786 563756 BD Horizon 1:100
Ly6C HK1.4 BV785 128041 Biolegened 1:80
Ly6G 1A8 BV711 127643 Biolegened 1:80
MHCII (I-A/I-E) M5/114.15.2 FITC 107605 BioLegend 1:800
PD-1 (CD279) 29F.1A12 BV785 135225 BioLegend 1:160
PD-L1 (CD274) 10F.9G2 PE 124308 BioLegend 1:80
TNF-α MP6-XT22 BV605 506329 BioLegend 1:50
CD16/32 2.4G2 - BD Biosciences AB_2687830 1:50

Wester blot
Anti-S6 54D2 2238583 Cell Signalling 1:1000
Anti-p-S6 (Ser235/236) Polyclonal 331679 Cell Signalling 1:1000
Anti-Erk1/2 3A7 10695739 Cell signalling 1:1000
Anti-p-Erk1/2 (Thr202/Tyr204) 9101 331646 Cell Signalling 1:1000
Anti-Akt 40D4 1147620 Cell Signalling 1:1000
Anti-p-Akt (Ser473) D9E 2315049 Cell Signalling 1:1000
Anti-Vinculin VIN-11-5 2877646 Sigma 1:2000

Spectral
CD45 30-F11 PerCP 103129 BioLegend 1:200
CD19 6D5 BV570 115535 BioLegend 1:200
CD11b M1/70 BV480 566117 BD Biosciences 1:400
NK1.1 PK136 PE/Cy5 108715 BioLegend 1:200
CD3 17A2 BUV805 569192 BD Biosciences 1:150
CD8α 53-6.7 Spark Blue 550 100779 BioLegend 1:200
CD4 GK1.5 APC/Fire 810 100479 BioLegend 1:200
FOXP3 FJK-16 eFluor660 50-5773-82 eBioscience 1:200
CD11c N418 BUV563 749040 BD Biosciences 1:150
Siglec-F S17007L PerCP/Fire 806 155535 BioLegend 1:200

	CD24 M1/69 FITC 101805 BioLegend 1:400<br>Ly6G 1A8 BV711 127643 BioLegend 1:200<br>CD206 C068C2 PE/Fire 700 141741 BioLegend 1:400<br>Ly6C HK1.4 BV785 128041 BioLegend 1:200<br>MerTK 2B10C42 PE/Dazzle 594 151523 BioLegend 1:150<br>CD64 X54-5/7.1 PE/Cy7 139313 BioLegend 1:200<br>MHCII M5/114.15.2 PerCP-eFluor710 46-5321-82 eBioscience 1:600<br>CD86 GL-1 AF-700 105023 BioLegend 1:400<br>CD103 2E7 BV605 121433 BioLegend 1:100<br>SIRPα P84 APC/Fire750 144029 BioLegend 1:200<br>CD49b DX5 BB700 568015 BD Biosciences 1:200<br>CD107α 1D4B BUV395 565533 BD Biosciences 1:150<br>PD-L1 10F.9G2 PE 124307 BioLegend 1:400<br>Arginase 1 A1exF5 eFluor450 48-3697-82 eBioscience 1:200<br>Ki-67 SolA15 BUV615 366-5698-82 eBioscience 1:600<br>TIM3 RMT2-23 BUV661 753149 BD Biosciences 1:100<br>CD62L MEL-14 BUV737 612833 BD Biosciences 1:200<br>CD44 IM7 BV421 103039 BioLegend 1:200<br>CD69 H1.2F3 BV510 104531 BioLegend 1:150<br>B220 RA3-6B2 BUV496 612950 BD Biosciences 1:400<br>PD-1 J43 BV650 569506 BD Biosciences 1:200<br>H-2Kb AF6-88.5.5.3 NovaFluorBlue610/70S 17836316 Invitrogen 1:200<br>CD71 C2 RB780 755614 BD Biosciences 1:400<br>CTLA-4 UC10-4B9 PE/Fire810 106335 BioLegend 1:150
Validation	Validation of antibodies available from commercial sources provided from: https://www.biolegend.com/, https://www.bdbiosciences.com/, https://www.thermofisher.com/uk/en/home/brands/invitrogen.html. Antibodies used have been extensively used in other studies.

Eukaryotic cell lines

Policy information about cell lines and Sex and Gender in Research

Cell line source(s)	3LL-ΔNRAS, derived from Lewis Lung Carcinoma, modified by NRAS knockout as described previously in Molina-Arcas et al., Sci. Transl. Med. 2019.<br>KPAR-G12C, derived from a KrasG12D/WT; Trp53fl/fl; Rosa26APOBEC3Bi/WT; Rag1KO/KO adeno-cre driven GEMM and prime edited as described in Boumelha et al., Cancer Research, 2022.<br>Human cell lines NCI-23 and Calu-1 have been provided by the Francis Crick Institute Cell Services facility.<br>KPB6-G12C, derived from a KrasG12D/WT; Trp53fl/fl; adeno-cre driven GEMM and CRISPR-KI as described in Mugarza et al., Science Advances, 2022.<br>KPAR.M7 was derived from KPAR-G12C orthotopic lung tumours growing on 50 mg/kg MRTX849 (Adagrasib).
Authentication	3LL-ΔNRAS and KPAR-G12C cell lines were sequenced by WES. DNA finger printing for human cell lines.<br>KPAR.M7 has not yet been authenticated.
Mycoplasma contamination	All cell lines were tested regularly for Mycoplasma and found negative.
Commonly misidentified lines<br>(See ICLAC register)	No commonly misidentified cell lines were used.

Animals and other research organisms

Policy information about studies involving animals; ARRIVE guidelines recommended for reporting animal research, and Sex and Gender in Research

Laboratory animals	C57BL/6J and RAG1-/- from a C57BL/6J background mice were used.
Wild animals	N/A
Reporting on sex	The KPARG12C cell line was generated from a female mouse whereas the 3LL-ΔNRAS cell line was derived from a male mouse. Our study examined antitumour immune responses generated by targeted therapy and to avoid introducing error due to induction of immune responses in female mice against genes found in the Y chromosome, we used mice with the same sex as the sex of the mouse that the cell lines were derived from, i.e. male mice for transplantation of 3LL-ΔNRAS cells and female mice for transplantation of KPARG12C cells. Similarly, KPAR.M7 were derived from KPARG12C tumours and therefore only female mice were transplanted with this cell line. Our study therefore examined both male and female animals, but in different settings.
Field-collected samples	N/A
Ethics oversight	All animal studies and procedures were performed in accordance to the Francis Crick Institute welfare guidelines, under a UK Home

Office-approved project license (P19FC0E42). All study plans were approved by Biological Research Facility at the Francis Crick Institute. All transplantation animal experiments were carried out using 8-10-week C57BL/6J mice. Mice housing is maintained with a 12-12 hour light-dark cycle and in specific-pathogen-free conditions with each cage individually ventilated and never exceeding 5 mice.Humidity and temperature are maintained according to UK Home Office guidelines, 20-24 °C and 45-65%, respectively.

Note that full information on the approval of the study protocol must also be provided in the manuscript.

Plants

Seed stocks	N/A
Novel plant genotypes	N/A
Authentication	N/A

Flow Cytometry

Plots

Confirm that:

- [x] The axis labels state the marker and fluorochrome used (e.g. CD4-FITC).
- [x] The axis scales are clearly visible. Include numbers along axes only for bottom left plot of group (a 'group' is an analysis of identical markers).
- [ ] All plots are contour plots with outliers or pseudocolor plots.
- [x] A numerical value for number of cells or percentage (with statistics) is provided.

Methodology

Sample preparation	Lung tumours were isolated from lungs using sterilised forceps. Multiple tumours from a single mouse were pooled together. Tumours were finely cut into small pieces and digested with collagenase (1 mg/ml; Thermo Fisher Scientific) and DNase I (50 U/ml; Life Technologies) in HBSS for 45 min at 37°C. Samples were filtered through 70 um strainers (Falcon) and red blood cells were lysed using ACK buffer (Life Technologies). Cell lines were grown under standard conditions and harvested by trypsinization.
Instrument	Fortessa Symphony A5.
Software	Data acquisition was done using BD FACSDiva Software v9.1. FlowJo 10.8.1 and Prism 10 were used for data analysis.
Cell population abundance	N/A. No sorting was performed.
Gating strategy	Gating Strategy for all immune populations is provided in Supplementary Figure 7

- [x] Tick this box to confirm that a figure exemplifying the gating strategy is provided in the Supplementary Information.
